# Lewy body co‐pathology in Alzheimer's disease and primary age‐related tauopathy contributes to differential neuropathological, cognitive, and brain atrophy patterns

**DOI:** 10.1002/alz.14191

**Published:** 2024-12-22

**Authors:** Francisco C. Almeida, Alexandra Santos, Tiago Jesus, Ana Coelho, Miguel Quintas‐Neves, Kathryn Gauthreaux, Charles N. Mock, Walter A. Kukull, John F. Crary, Tiago Gil Oliveira

**Affiliations:** ^1^ Life and Health Sciences Research Institute (ICVS) School of Medicine University of Minho, Campus Gualtar Braga Portugal; ^2^ ICVS/3B's—PT Government Associate Laboratory Braga Portugal; ^3^ Department of Neuroradiology Centro Hospitalar Universitário do Porto Porto Portugal; ^4^ Center Algoritmi LASI, University of Minho, Campus Gualtar Braga Portugal; ^5^ Department of Neuroradiology Hospital de Braga, ULS Braga Braga Portugal; ^6^ Department of Epidemiology National Alzheimer's Coordinating Center University of Washington Seattle Washington USA; ^7^ Neuropathology Brain Bank & Research Core, Department of Pathology, Nash Family Department of Neuroscience, Department of Artificial Intelligence & Human Health, Friedman Brain Institute Ronald M. Loeb Center for Alzheimer's Disease, Icahn School of Medicine at Mount Sinai New York New York USA

**Keywords:** Alzheimer's disease, co‐pathology, Lewy bodies, MRI, primary age‐related tauopathy

## Abstract

**INTRODUCTION:**

Alzheimer's disease (AD) co‐pathology with Lewy bodies (LB) is frequent and influences clinical manifestations and outcomes. Its significance in primary age‐related tauopathy (PART) is unknown. We investigated the influence of LB on cognition and brain atrophy in AD and PART.

**METHODS:**

We performed a retrospective cohort study in a large sample of autopsied participants with AD neuropathological change (ADNC) with and without LB and PART with and without LB, with corresponding *ante mortem* magnetic resonance imaging (MRI) data from the National Alzheimer's Coordinating Center dataset.

**RESULTS:**

LB co‐pathology worsened cognitive impairment in both PART and ADNC. On longitudinal follow‐up, LB impacted cognitive decline in multiple domains. Additionally, LB influenced brain atrophy on MRI across groups and LB regional staging was different in PART and ADNC, accompanying tauopathy progression.

**DISCUSSION:**

These results suggest that LB co‐pathology is associated with divergent patterns of cognitive impairment, brain atrophy, and regional pathological distribution in PART and AD.

**Highlights:**

- Lewy body (LB) co‐pathology is frequent in Alzheimer's disease (AD) with important clinical implications.
- LB co‐pathology is also present in primary age‐related tauopathy (PART), but its significance is still understudied.
- In PART and AD, LB leads to higher cognitive impairment and brain regional atrophy.
- In PART and AD, LB tends to accompany neurofibrillary tangle progression, suggesting amyloid pathology might be a trigger for regional pathology progression.

## 1 BACKGROUND

Alzheimer's disease (AD) is characterized by the deposition of extracellular amyloid‐beta (Aβ) neuritic plaques (NPs) and hyperphosphorylated tau in the form of neurofibrillary tangles (NFTs).^1^ Recently, autopsy cases revealed NFT deposition predominantly confined to the entorhinal and the medial temporal cortex, the typical topography of early stages of AD, in the absence of Aβ NPs. This neuropathology phenotype has been called primary age‐related tauopathy (PART).^2^ Since its emergence, there has been debate on whether PART is a separate nosological entity from AD^3^ or represents an initial phase within the AD spectrum.^4^

Independently of a potentially distinct pathophysiology between AD and PART, various studies addressing the *ante mortem* features of *post mortem* confirmed cases, highlighted differences at the clinical, imaging, neuropathology, and genetic levels. PART patients can present with multiple domain mild cognitive impairment (MCI) or dementia, although usually less severe and with a slower progression over time compared with AD.^5^, ^6^, ^7^, ^8^, ^9^ In PART, as in AD, cognitive impairment correlates with the burden and extension of tau pathology,^5^, ^10^, ^11^, ^12^, ^13^, ^14^, ^15^ suggesting NFTs are likely a contributing factor. On magnetic resonance imaging (MRI) of PART patients, Braak stage correlates with left hippocampal head atrophy^10^ and anterior temporal regional atrophy correlates with lower scores on memory/fluency and language tests.^15^, ^16^ Interestingly, when compared with AD, PART showed less atrophy only in the medial temporal lobe and only when compared with Consortium to Establish a Registry for Alzheimer's Disease (CERAD 3) scores, which represent advanced cases of neuritic plaque pathology.^15^ This indicates that PART brings about a degree of neurodegeneration comparable to the initial stages of AD. Moreover, the topography of hyperphosphorylated tau deposition within the hippocampus is different in AD and PART, with higher deposition in the CA2 region in the latter.^17^, ^18^ Finally, the *apolipoprotein E (APOE)* allele ε2 has been shown to be more frequent in PART than in AD,^5^, ^8^, ^19^ which could be contributing to the differential phenotypes observed in PART, since the *APOE* allele ε2 has been shown to confer protection, while ε4 is associated with higher risk in AD.^20^ Overall, these findings indicate that there are discrepancies in the presentation of these two groups, from clinical manifestations to genetics, underpinning the need to diagnose these pathologies in vivo, especially when treatment considerations based on Aβ deposition are starting to enter routine clinical practice.^21^ Furthermore, it was previously reported that around 50% of patients with PART are diagnosed during life as clinical AD,^22^ thus emphasizing the importance of studying biomarkers to separate these two entities. Even though MRI is an essential step in the routine clinical evaluation of cognitive impairment, the patterns of cortical atrophy in AD and PART have only been scarcely addressed.

Besides the diagnostic hallmarks of AD, such as NPs and NFTs, patients often present with additional co‐pathologies.^23^ In particular, Lewy body (LB) pathology is frequent in AD neuropathological assessments, and AD pathology is often detected in probable LB dementia (LBD), with implications to clinical manifestations, biomarker cutoffs, and prognosis.^23^, ^24^, ^25^, ^26^, ^27^, ^28^, ^29^, ^30^, ^31^, ^32^, ^33^, ^34^, ^35^, ^36^ PART also presents with other pathologies. Indeed, TAR DNA binding protein 43 (TDP‐43) is relatively frequent in this subgroup and correlates with hippocampal, amygdala, and anterior temporal atrophy, a finding that is even stronger in AD patients.^14^, ^37^, ^38^ Although tauopathy without amyloidosis is known to occur in patients with LB,^25^, ^28^, ^39^ its implications for the disease are not well studied as this group appears to be uncommon. This is relevant since it might reveal possible protein contributions to cognitive impairment and neurodegeneration among these mixed‐pathology groups.

Here, leveraging the large National Alzheimer's Disease Coordinating Center (NACC) dataset, which includes autopsy data from longitudinally followed dementia patients, we compared *ante mortem* clinical dementia severity scores and MRI cortical and subcortical atrophy between AD neuropathological change (ADNC) with and without LB and PART with and without LB.

## 2 METHODS

### 2.1 Participants

Data were obtained from the NACC, a repository for data collected at the AD Centers (ADCs) across the United States of America. The ADCs collect standardized clinical data via the Uniform Data Set (UDS) and neuropathological evaluations obtained at autopsy from patients who consented to the Neuropathology Data Set. The UDS and Neuropathology Data Set data have been described before.^40^, ^41^, ^42^, ^43^, ^44^

### 2.2 Selection criteria

A flowchart depicting the selection process of the cohorts included in this research is available in Figure S1. Utilizing the March 2024 NACC data freeze, 50,259 participants were included. Of these, 7595 participants had available CERAD scores, Braak stage, and LB stage. From this cohort, our MRI sample was obtained after excluding those participants without ADNC pathology (CERAD score of none and Braak stage 0), with neuropathological or clinical evidence of 3R or 4R tauopathy, frontotemporal degeneration, argyrophilic grain disease, amyotrophic lateral sclerosis, prion disease, multiple system atrophy, and Parkinson's disease, clinical UDS data more than 2 years from death, non‐volumetric T1‐weighted images or brain lesions that biased atrophy assessment (e.g., brain tumor, brain herniation, vascular malformation, lymphocytic meningoencephalitis, traumatic brain injury, demyelinating disease). The sample for MRI analyses consisted then of 322 participants. The neuropathological sample was obtained from the 7595 participants after excluding those participants without ADNC pathology, neuropathological or clinical evidence of other neurodegenerative diseases (as cited above), and without distinction of amygdala‐predominant and limbic LB pathology (no “NPLBOD” scores available), totalizing 3413 participants for neuropathology analyses. For the cross‐sectional cognitive analyses, out of the 3413, 1955 participants had the last UDS visit < 2 years before death. In the longitudinal cognitive analysis, patients with only one data point were excluded, totaling 1808 participants for neuropsychological analyses.

### 2.3 Neuropathology data

Participants were categorized according to the Braak stage for NFT degeneration, CERAD score for NPs, and the presence of LB inclusions. Throughout, the LB stage was defined according to the “NPLBOD” variable in the NACC: olfactory bulb, brainstem, limbic/transitional (limbic), amygdala‐predominant, and neocortical/diffuse (neocortical). Presence of limbic‐predominant age‐related TDP‐43 encephalopathy neuropathologic changes (LATE‐NC), categorized as TDP‐43 inclusions in the amygdala, hippocampus, and/or entorhinal cortex, according to,^45^ presence of age‐related tau astrogliopathy (ARTAG) and cerebral amyloid angiopathy (CAA). Details on brain tissue preparation and staining within the NACC neuropathology dataset have been previously described.^44^ ADNC was defined as the presence of NPs as assessed by the CERAD scores with or without NFT pathology as assessed by the Braak stage.^46^ PART was defined as the presence of NFTs in the absence of NPs.^2^

**RESEARCH IN CONTEXT**

1. **Systematic review**: Alzheimer's disease (AD) is often accompanied by Lewy body (LB) co‐pathology with implications for clinical symptoms and outcomes. Primary age‐related tauopathy (PART) also presents with LB, but its significance has not been studied.
2. **Interpretation**: Our findings suggest that LBs in PART and AD play an important role in cognitive impairment and patterns of brain atrophy. LBs tend to accompany tauopathy stage progression in PART and AD, suggesting amyloid‐beta might ignite the movement to the neocortex of both proteinopathies in mixed‐pathology cases.
3. **Future directions**: Longitudinal assessment of LB and tau pathology progression in PART and AD might provide critical insights into the mechanisms of co‐pathology and their clinical significance.

### 2.4 Brain MRI data

The MRI analyses were performed on 1.5T or 3T scanners, both from Philips, Siemens, or GE manufacturers. Data were provided by the NACC. For our imaging analyses, we used volumetric T1‐weighted acquisitions.

### 2.5 Image analysis

We used Freesurfer software v7.2 to calculate brain regional volumes from T1‐weighted volumetric images.^47^ For cortical parcellations we used the Desikan–Killiany atlas^48^ and for subcortical segmentation the Aseg atlas.^49^ All regional volumes were divided by intracranial volume. Brain plots were obtained using the *ggseg* package from R.^50^

### 2.6 Cross‐sectional and longitudinal cognitive measurements

Local ADCs assessed participants using the Cognitive Dementia Rating Scale (CDR) and the UDS version 2 neuropsychological test battery.^42^ The Washington University CDR was reviewed by Morris and collaborators in 1993^51^ with the purpose of staging the severity of AD and takes into consideration the following six cognitive categories: memory, orientation, judgment and problem‐solving, community affairs, home and hobbies, and personal care. The CDR Sum of Boxes (CDR‐SB) is calculated by summing the ratings of the six cognitive domains, ranging from 0 (normal) to 18 (severe dementia).^52^ For cross‐sectional analysis, CDR‐SB was collected at the last visit before death. Groups were divided into three dementia severity groups based on CDR global scores: 0, normal cognition (NC); 0.5, MCI; > 0.5, dementia (Dem). For longitudinal analysis, a retrospective follow‐up period of 2000 days was selected. Executive function was assessed by the Trail Making Test (TMT) A and B, which globally tests attention, visual scanning and search skills, and psychomotor speed and coordination^53^; TMT A can independently assess processing speed, while TMT B assesses set switching; on both parts of this test (i.e., A and B), the total number of seconds to complete the test, the number of commission errors, and number of correct lines were recorded; the Wechsler Adult Intelligence Scale digit symbol (WAIS) test was also considered to provide an estimate of processing speed.^54^ Semantic memory/language was assessed by category (vegetables and animals) verbal fluency,^55^ consisting of a test on registering the total number of vegetables and animals named in 60 s; the Boston naming test,^56^ which also assesses the effect of language function, more precisely the confrontational word retrieval, was included in this evaluation and consisted of showing pictures (up to 60) to the patient, and wait up to 20 s for the patients to name them. Attention and working memory were evaluated by Digit Span Forwards (DIGIF) and Backwards test (DIGIB),^57^ consisting of registering the ability to recall a sequence of numbers shown, and the total length of numbers successfully achieved (DIGIFL and DIGIBL, respectively). Logical memory was evaluated using Logical Memory Immediate and Delayed Recall tests (Logical Memory and Memory Unit), in which an orally presented verbal story is asked to be recalled immediately and 20 minutes later.^58^ Mini‐Mental State Examination (MMSE)^59^ was performed as a brief cognitive screening instrument that provides a measure of global cognition.

### 2.7 Statistical analysis

Data normality was assessed using Kolmogorov–Smirnov tests. The homogeneity of variance was tested using Levene's test. To assess potential differences between groups in terms of demographic characteristics, Welch's analysis of variance (ANOVA) was followed by Tukey honestly significant difference (HSD) for multiple comparisons, Kruskall–Wallis test followed by Dunn's test with Holm correction for multiple comparisons or chi‐squared test or Fisher's test with Holm correction was performed, as appropriate. For longitudinal analysis of cognitive and neuropsychological tests, a multiple linear mixed‐effects regression model was used with the test as the dependent variable, group, and group*time since last visit interaction as independent variables and with random slopes for elapsed time and intercepts per subject. The signal of time since the last visit was inverted for ease of interpretation of beta‐coefficients. Whenever missing data were present, subjects were excluded from the analysis. We used the *lmerTest* package in R to perform the regression and calculate *p *values.^60^ The package *emmeans* was used for marginal slope comparison. For volumetric analyses, linear regression with volume as the dependent variable and age at MRI, or age at MRI and Braak stage, as the independent variables was calculated. Residuals were then obtained, *z*‐scored, and Welch's ANOVA or Kruskall–Wallis test was used to compare the groups. False discovery rate correction (FDR) was applied to correct for multiple comparisons across brain regions (statistical significance considered for *p* < 0.05 after correction). When statistically significant findings were detected after FDR, Tukey HSD or Dunn's test was used for pairwise comparisons. All analyses and figures were created using RStudio Version 1.4.1103.

## 3 RESULTS

### 3.1 Demographics and neuropathological characteristics

Out of 7595 participants with available Braak stage, CERAD score, and LB stage of any type, ADNC without LB (ADNC w/o LB) was the most frequent group, accounting for 40.2%, ADNC with LB (ADNC+LB) for 26.4%, PART without LB (PART w/o LB) for 9.0%, and PART with LB (PART+LB) for 2.6%, with other pathologies (see Figure S1 and Methods for details) accounting for 21.8% (Figure S2).

For a detailed neuropathological analysis, we next included only participants with LB stage neuropathology that distinguished “amygdala‐predominant” from the limbic stage and excluded participants with no ADNC pathology and with other neuropathologies (Figure S1). This cohort was composed of 3413 participants. Summary statistics for demographic comparisons across groups are shown in Table 1 and pairwise comparisons are in Table S1. ADNC+LB and PART+LB had a higher proportion of male participants compared with the respective groups without LB (*p* < 0.05 with chi‐squared test after Holm correction). ADNC+LB had a significantly earlier age at death compared with the other groups. ADNC w/o LB and PART+LB had an earlier age at death compared with PART w/o LB (*p* < 0.05 with Dunn's test after Holm correction).

**TABLE 1:** Demographics and neuropathology of ADNC and PART patients with and without LB.

Cohort (*n* = 3413)	PART w/o LB	PART+LB	ADNC w/o LB	ADNC+LB	*p*‐value
Sample size	394	123	1615	1281	
Male (%)	198 (50.2%)	79 (64.2%)	748 (46.3%)	671 (52.4%)	***X*(3) = 21.51; *p* < 0.001**
Age at death (IQR)	88 (11)	86 (14)	85 (15)	80 (15)	***H*(3) = 164.23; *p* < 0.001**
Braak stage (%)					
0	0 (0.0%)	0 (0.0%)	5 (0.3%)	3 (0.2%)	ADNC: *p* = 0.19^a^
I–II	242 (61.4%)	63 (51.2%)	110 (6.8%)	56 (4.4%)	PART: *p* = 0.19^b^
III–IV	148 (37.6%)	55 (44.7%)	362 (22.4%)	179 (14.0%)	
V–VI	4 (1.0%)	5 (4.1%)	1138 (70.5%)	1043 (81.4%)	
CERAD score (%)					
None			0 (0.0%)	0 (0.0%)	ADNC: *X*(2) = 3.91; *p* = 0.14
Mild			245 (15.2%)	105 (8.2%)	
Moderate			398 (24.6%)	246 (19.2%)	
Severe			972 (60.2%)	930 (72.6%)	
LB stage (%)					
Olfactory bulb		5 (4.1%)		58 (4.5%)	
Brainstem		29 (23.6%)		89 (6.9%)	
Limbic		34 (27.6%)		263 (20.5%)	***p* < 0.001** ^c^
Amygdala‐predominant		9 (7.3%)		461 (36.0%)	
Neocortical		46 (37.4%)		410 (32.0%)	

*Note*: The proportion of male sex and age at death differed between groups. See Table S1 for pairwise comparisons.

Bold values indicate statistical significance.

^a^ADNC+LB Braak stage proportions compared to ADNC w/o LB using Fisher's test was not statistically significant.

^b^PART+LB Braak stage proportions compared to PART w/o LB using Fisher's test was not statistically significant.

^c^ADNC+LB LB stage proportions compared to PART+LB using Fisher's test were statistically significant.

Abbreviations: ADNC, Alzheimer's disease neuropathological change; ADNC+LB, ADNC with LB, ADNC w/o LB, ADNC without LB; LB, Lewy bodies; PART w/o LB, PART without LB; PART+LB, PART with LB; PART w/o LB, PART without LB; PART, primary age‐related tauopathy; PART+LB, PART with LB.

Braak stage and CERAD score proportions were not significantly different in ADNC w/o LB and ADNC+LB. Braak stage proportions were not significantly different in PART w/o LB versus PART+LB. The distribution of LB stages was different in ADNC+LB compared to PART+LB, with a higher proportion of amygdala‐predominant cases in ADNC+LB (Table 1). LATE‐NC, CAA, and ARTAG pathology proportions in cases with available data are shown in Table S2. Groups differed in the proportion of CAA and LATE‐NC co‐pathologies, with pairwise comparisons showing a higher proportion of LATE‐NC in ADNC+LB compared with PART w/o LB and PART+LB, and a higher proportion of CAA in ADNC+LB and ADNC w/o LB compared with both PART w/o LB and PART+LB (*p*‐value < 0.05 with chi‐squared tests after Holm correction) (Table S2).

### 3.2 LB in PART and ADNC associated with cognitive impairment

**FIGURE 1:**
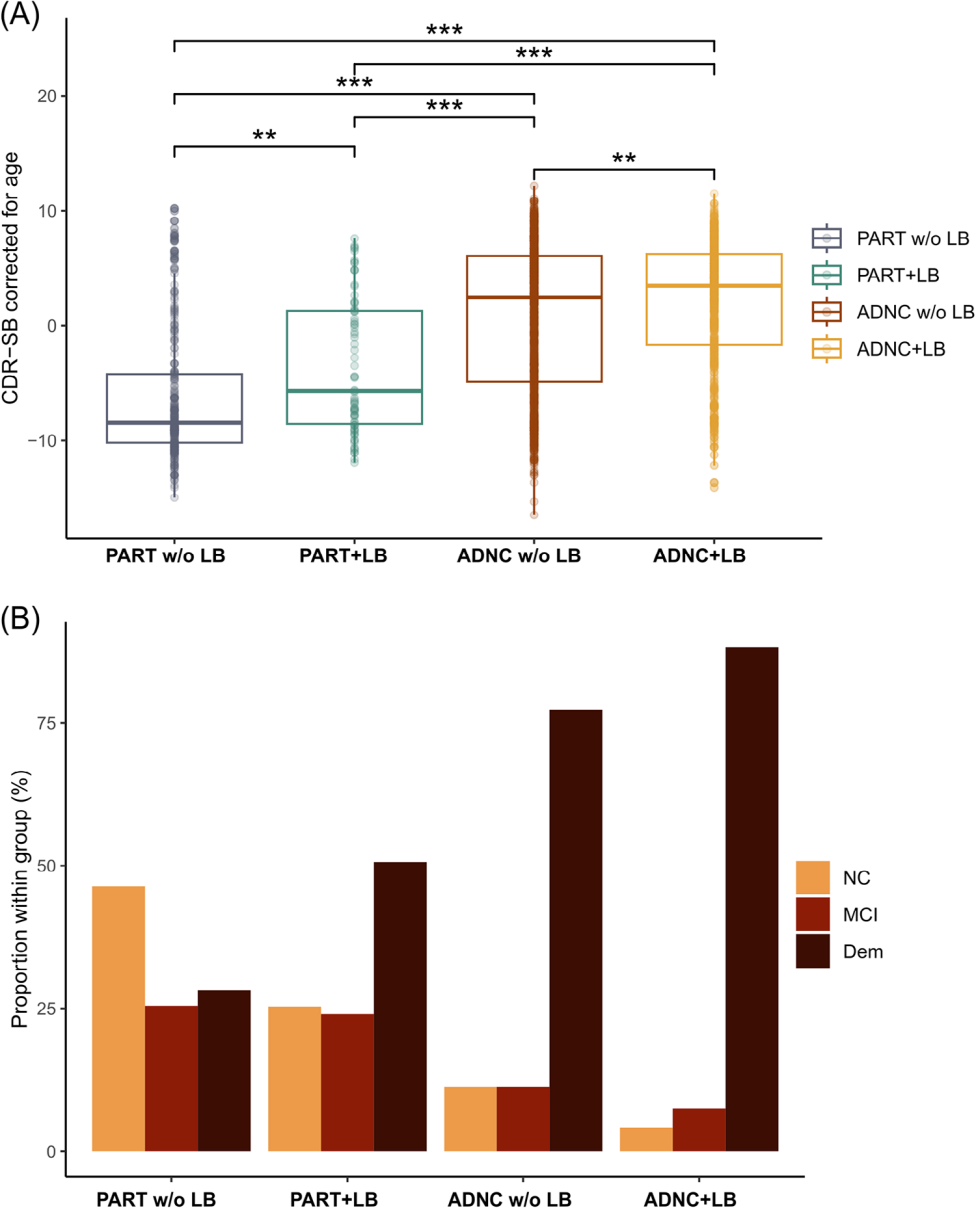
The presence of LB associates with cognitive impairment in PART. (A) The comparison of CDR‐SB residuals after linear regression with age across the four groups. ADNC+LB showed the highest dementia severity followed by ADNC w/o LB, PART+LB and PART w/o LB, with all pairwise comparisons statistically significant (*p < 0.05; **p < 0.01; ***p < 0.001, with Dunn's test after Holm correction). (B) The proportion of dementia severity based on CDR global shows the same gradient across groups. All pairwise comparisons were statistically significant (*p* < 0.05 with chi‐squared test after Holm correction). Sample size, *n* = 1955 (PART w/o LB, *n* = 263; PART+LB, *n* = 75; ADNC w/o LB, *n* = 953; ADNC+LB, *n* = 664). ADNC, Alzheimer's disease neuropathological change; ADNC+LB, ADNC with LB; ADNC w/o LB, ADNC without LB; CDR, Cognitive Dementia Rating Scale; Dem, dementia; LB, Lewy bodies; MCI, mild cognitive impairment; NC, normal cognition; PART w/o LB, PART without LB; PART, primary age‐related tauopathy; PART+LB, PART with LB.

Out of the neuropathological cohort, 1955 participants had a UDS visit less than 2 years before death (PART w/o LB, *n* = 263; PART+LB, *n* = 75; ADNC w/o LB, *n* = 953; ADNC+LB, *n* = 664). Cross‐sectionally, ADNC+LB presented with the highest median CDR‐SB score corrected for age, followed by ADNC w/o LB and PART+LB and PART w/o LB. All pairwise comparisons were statistically significant (*p* < 0.05 with Dunn's test after Holm correction; Figure 1A). The proportion of dementia severity based on CDR global scores (NC, MCI, and Dem) also differed significantly between all groups in the same gradient of dementia severity (*p* < 0.001 for all pairwise comparisons with chi‐squared test after Holm correction; Figure 1B). Even though the proportion of Braak stages did not differ significantly within PART and ADNC groups, the groups with co‐pathology showed a higher proportion of more advanced Braak stages. In order to test whether differences in cognitive impairment within PART and ADNC were due to Braak stage imbalances, we corrected CDR‐SB for age and Braak stage and compared PART w/o LB with PART+LB and ADNC w/o LB with ADNC+LB. Wilcoxon's tests showed a statistically significant lower CDR‐SB score in PART w/o LB (median residuals, ‐2.53; interquartile range [IQR], 5.42) compared with PART+LB (median residuals, ‐0.33; IQR, 9.94; *p* < 0.001). Division according to Braak stage within PART groups also showed statistically significant differences between PART w/o LB and PART+LB within the same Braak stage (Figure S3A). ADNC w/o LB also showed a lower CDR‐SB score compared with ADNC+LB after age and Braak correction (ADNC w/o LB—median residuals, 0.95; IQR, 8.84; ADNC+LB—median residuals, 2.10; IQR, 6.37; *p* < 0.009, using Wilcoxon's test). Division according to Braak stage within ADNC groups showed statistically significant differences between ADNC w/o LB and ADNC+LB in Braak III–IV stages, but not on I–II or on V–VI, suggesting a possible ceiling effect as measured by the CDR‐SB (Figure S3B).

We next tested the longitudinal progression of cognitive impairment with a battery of neuropsychological tests (Figure S4 and Tables S3–5). Using multiple linear regression mixed‐effects models, ADNC+LB and ADNC w/o LB progressed faster in cognitive impairment in all neuropsychological tests compared to PART w/o LB. PART+LB progressed faster compared with PART‐LB in CDR‐SB and TMT‐A. ADNC w/o LB declined faster in CDR‐SB, MMSE, and Boston Naming Test compared with PART+LB. ADNC+LB declined faster compared with PART+LB in CDR‐SB, MMSE, WAIS, Animals, Vegetables, Boston Naming Test, TMT‐A, TMT‐B, and DIGIF‐L and Logical Memory and faster than ADNC w/o LB in CDR‐SB, MMSE, WAIS, Animals, Boston Naming Test, TMT‐A, DIGIF, and DIGIF‐L. These results suggest a worse progression in ADNC+LB compared with other groups across multiple cognitive domains, followed by ADNC w/o LB, PART+LB, and PART‐LB.

### 3.3 LB in PART and ADNC are associated with differential patterns of brain atrophy

**FIGURE 2:**
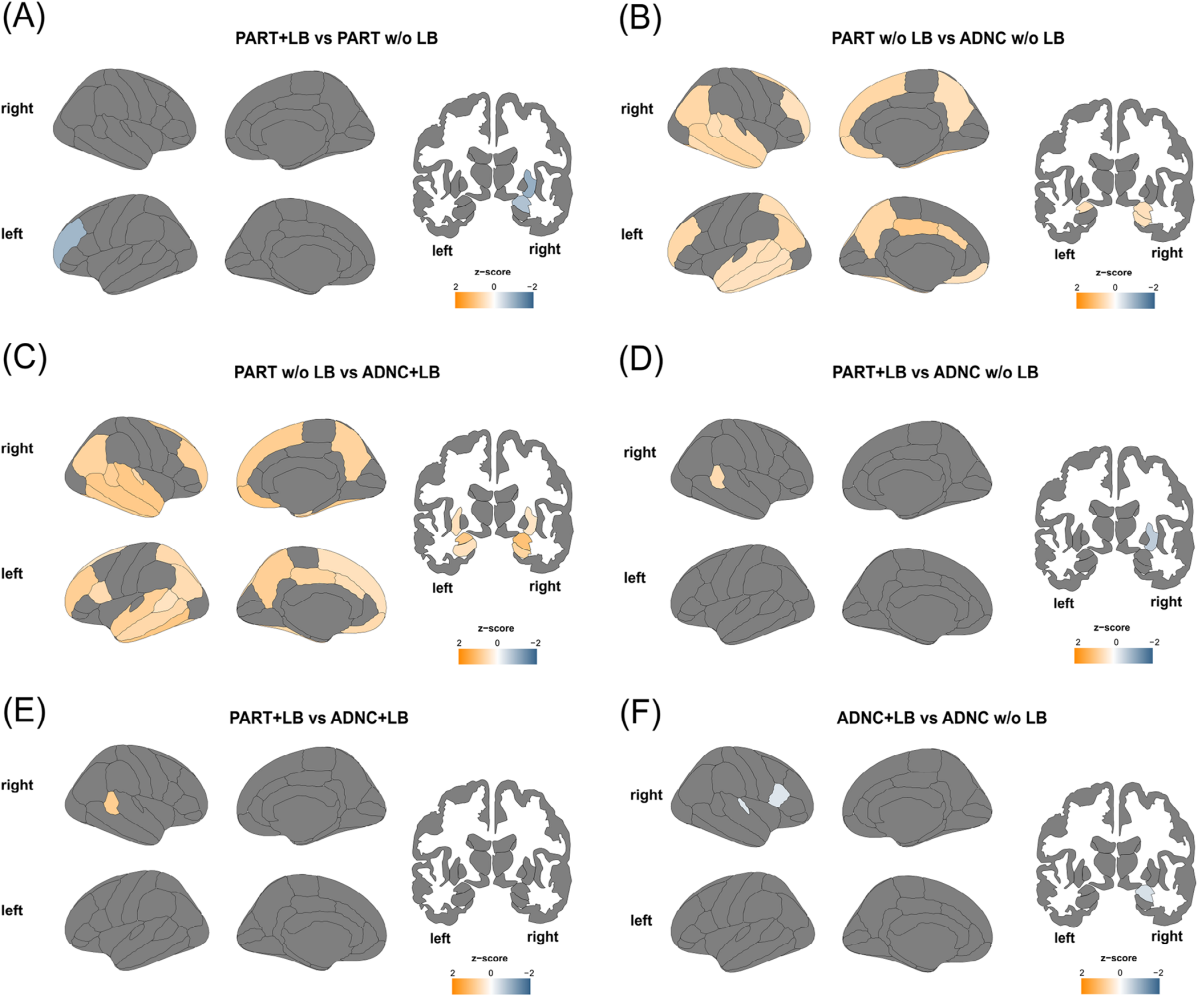
The presence of LB is associated with higher regional atrophy ADNC and PART. This figure shows the comparison of cortical and subcortical volumes for ADNC and PART with and without LBs. *z*‐scores based on mean difference in brain volume residuals after linear regression with age at MRI are shown in the brain graph in (A) PART+LB versus PART w/o LB; (B) PART w/o LB versus ADNC w/o LB; (C) PART w/o LB versus ADNC+LB; (D) PART+LB versus ADNC w/o LB; (E) PART+LB versus ADNC+LB; (F) ADNC+LB versus ADNC w/o LB. The presence of LB pathology was associated with lower cortical and subcortical volume in both ADNC and PART, whereas PART w/o LB pathology showed higher volumes in widespread cortical and subcortical areas compared with ADNC w/o LB and ADNC+LB. Blue regions represent lower volumes for the first group in the comparison, whereas yellow represents higher volumes. Only significantly different comparisons are shown (*p* < 0.05 after FDR correction for Welch's ANOVA or Kruskall–Wallis Test and *p* < 0.05 after TukeyHSD or Dunn's test with Holm correction). When not significant, group comparisons are not shown. Sample size, *n* = 322 (PART w/o LB, *n* = 38; PART+LB, *n* = 13; ADNC w/o LB, *n* = 157; ADNC+LB, *n* = 114). ADNC, Alzheimer's disease neuropathological change; ADNC+LB, ADNC with LB; ADNC w/o LB, ADNC without LB; FDR, false discovery rate; LB, Lewy bodies; MRI, magnetic resonance imaging; PART w/o LB, PART without LB; PART, primary age‐related tauopathy; PART+LB, PART with LB.

ADNC and PART present with preferential deposition of NFTs along a well‐described brain topographical pattern described by the Braak stage progression in AD. This raises the hypothesis that cortical atrophy in PART should be limited up to the medial temporal lobe when compared to AD. Moreover, the hippocampus and amygdala are subcortical structures well known to be affected in both ADNC and PART. How the concomitance of LB might contribute to atrophy is still to be elucidated. After exclusion criteria (Figure S1), the cohort was composed of 322 participants (PART w/o LB, *n* = 38; PART+LB, *n* = 13; ADNC w/o LB, *n* = 157; ADNC+LB, *n* = 114). We compared cortical and subcortical volumes between the four groups after obtaining residuals from linear regression with age (Figure 2).

PART+LB presented a lower volume of the left rostral middle frontal, right putamen, and right amygdala compared with PART w/o LB (*p* < 0.05 after FDR correction; Figure 2A).

ADNC w/o LB showed lower volume of multiple temporal (left fusiform, banks of the superior temporal sulcus, inferior, middle and superior temporal gyri; and right fusiform, banks of the superior temporal sulcus, inferior, middle, and superior temporal gyri), parietal (left inferior and superior parietal, precuneus gyri; and right precuneus and inferior parietal gyri), frontal (left and right rostral middle and medial orbitofrontal; and right superior gyri), left posterior and caudal anterior cingulate regions and higher atrophy of the amygdala bilaterally and right hippocampus when compared with PART w/o LB (*p* < 0.05 after FDR correction; Figure 2B).

A slightly different and more widespread pattern of volumetric differences was found for ADNC+LB compared with PART w/o LB, including lower volume of temporal (left fusiform, banks of the superior temporal sulcus, inferior, middle, superior, and transverse temporal gyri; and right entorhinal, fusiform, banks of the superior temporal sulcus, inferior, middle, superior, and transverse temporal gyri), parietal (left precuneus, inferior and superior gyri; and right precuneus and inferior gyri), frontal (left pars opercularis, rostral middle, superior, medial orbitofrontal gyri; and right medial orbitofrontal, rostral middle and superior frontal gyri), left posterior and caudal anterior cingulate and higher atrophy of the amygdala, hippocampus, and putamen bilaterally (*p* < 0.05 after FDR correction; Figure 2C).

ADNC w/o LB showed lower volumes in the left banks of the superior temporal sulcus compared with PART+LB, but higher volumes of the right putamen (*p* < 0.05 after FDR correction; Figure 2D). ADNC+LB only showed lower volumes in the left banks of the superior temporal sulcus compared with PART+LB (*p* < 0.05 after FDR correction; Figure 2E).

ADNC+LB presented higher atrophy of the right pars opercularis, transverse temporal gyri, and right amygdala compared with ADNC w/o LB (*p* < 0.05 after FDR correction; Figure 2F).

To correct for the potential effects of the Braak stage in these comparisons, we obtained brain regional volume residuals after linear regression with age and Braak stage. With this correction, the only statistically significant differences after FDR correction were decreased right amygdala volume in ADNC+LB compared with ADNC w/o LB and decreased right putaminal volume in PART+LB compared with PART w/o LB and ADNC w/o LB (Figure S5).

### 3.4 ADNC+LB and PART+LB show different topographical patterns of LB deposition

**FIGURE 3:**
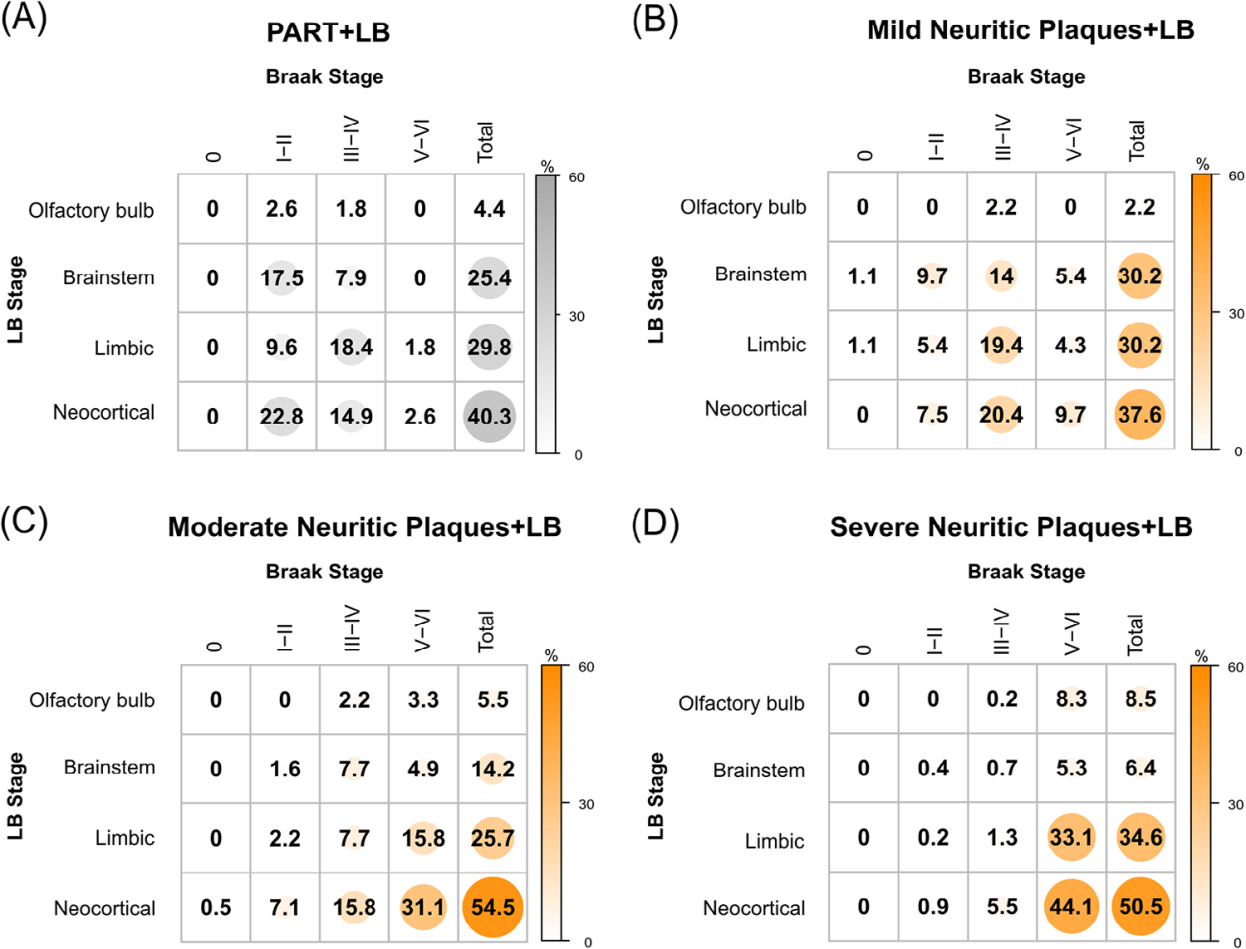
ADNC+LB and PART+LB show different topographical patterns of LB deposition. This figure shows the proportion of participants according to LB location and Braak stage in (A) PART+LB; (B) Mild NP+LB; (C) Moderate NP+LB; and (D) Severe NP+LB. In PART+LB and mild NP+LB, most patients located in early tau Braak stages (I–IV) and balanced LB stages, whereas in moderate to severe NP+LB, most patients localized to neocortical tau Braak stages (V–VI) and limbic and neocortical LB stages. Sample size, *n* = 1955 (PART w/o LB, *n* = 263; PART+LB, *n* = 75; ADNC w/o LB, *n* = 953; ADNC+LB, *n* = 664). ADNC, Alzheimer's disease neuropathological change; ADNC+LB, ADNC with LB; ADNC w/o LB, ADNC without LB; LB, Lewy bodies; NP, neuritic plaques; PART w/o LB, PART without LB; PART, primary age‐related tauopathy; PART+LB, PART with LB.

Given the differential distribution of NFTs in the PART and ADNC groups, we inspected the anatomical location of LBs in these groups to test whether LB pathology accompanies NFT pathology progression between PART and ADNC using the neuropathology cohort with 1955 participants. The distribution of LB pathology was significantly different between PART+LB and ADNC+LB (Table 1), mainly driven by the presence of “amygdala‐predominant” LB pathology in ADNC+LB. Given that this group is mainly localized to the amygdala, not following typical LB progression,^61^ we excluded it from this analysis, in order to test whether increasing amounts of NPs associated with the progression of LB pathology along with NFT pathology (Figure 3). A higher proportion of neocortical LB pathology and lower brainstem LB pathology was observed in Moderate NPs+LB (54.5% and 14.2%, respectively) and Severe NPs+LB (50.5% and 6.4%, respectively) compared to PART+LB (40.3% and 25.4%, respectively) and Mild NPs+LB (37.6% and 30.2%, respectively). Pairwise Fisher's tests with Holm correction were used to compare total LB distribution (sum of NFT Braak stages) across PART+LB and the different CERAD scores in ADNC+LB. PART+LB was not significantly different from Mild NPs+LB (*p* = 0.75). Both Moderate NPs+LB and Severe NPs+LB differed significantly from PART+LB and Mild NPs+LB (Moderate NPs+LB vs. PART+LB, *p *= 0.048; Moderate NPs+LB vs. Mild NPs+LB, *p *< 0.01; Severe NPs+LB vs. PART+LB, *p* < 0.001; Severe NPs+LB vs. PART+LB, *p* < 0.001). Severe NPs+LB also differed significantly from Moderate NPs+LB (*p* < 0.01).

## 4 DISCUSSION

In this study, we compared clinico‐demographic, neuropathological, and brain atrophy profiles between participants with ADNC and PART with or without LBs. We found differential patterns of cognitive impairment, brain atrophy pathology regional localization across the PART‐ADNC spectrum, and the absence/presence of LB co‐pathology.

The presence of LBs in ADNC is associated with earlier age of death, faster progression of cognitive impairment across multiple domains, and atrophy of frontal and temporal lobe and amygdala regions. Neocortical alpha‐synuclein inclusions were common in the ADNC+LB group, thus raising the hypothesis that in the most developed stages of the disease, this proteinopathy can act with tau to result in more severe neurodegeneration. Indeed, interactions between tau and alpha‐synuclein are known to occur and might act synergistically.^62^, ^63^, ^64^ Previous studies have shown that patients solely with LB pathology tend to show lower atrophy compared with AD, AD co‐pathology in LBD associates with higher atrophy compared with pure LBD, and ADNC compared to ADNC+LB shows mostly comparable or higher degrees of atrophy.^65^, ^66^, ^67^, ^68^, ^69^, ^70^, ^71^ These previous results suggest ADNC is the primary atrophy driver in co‐pathology with LB. However, differences in the burden of ADNC are likely to contribute to the diversity of atrophy patterns. Our sample was balanced for the Braak stage in the ADNC groups and results were corrected for the Braak stage, therefore suggesting that LB pathology might play a differentiating role in brain atrophy, especially of the amygdala.

Importantly, higher atrophy of the right amygdala was found in ADNC+LB versus ADNC w/o LB, even after correction for the Braak stage. This region is a known locus of co‐occurring pathology, thus supporting the notion that atrophy might be a marker of the interaction of these proteinopathies in this region.^72^ Further studies are needed to address how neurodegeneration of these regions might be related to regional susceptibility and specific cognitive deficits and clinical presentation in mixed pathology.

PART w/o LB represented a more benign group regarding dementia severity and this is reflected in a pattern of lower atrophy compared with the other groups. The presence of LBs in PART resulted in significant cognitive impairment and a pattern of atrophy localizing to the frontal lobe, the putamen, and the amygdala. The effect on the putamen was maintained when compared to ADNC w/o LB, but not compared to ADNC+LB. Interestingly, higher atrophy of the putamen had previously been shown in LBD compared with AD,^73^ suggesting a role of LB pathology in atrophy of the basal ganglia independent of AD pathology. To our knowledge, this is the first study to show a differential pattern of atrophy according to the presence of LBs in PART. Overall, these findings support the concept that LB co‐pathology is a determining factor of disease severity and degeneration in PART.

ADNC w/o LB and ADNC+LB showed a widespread pattern of higher cortical and subcortical atrophy, which ranged from the hippocampus to the frontal lobe, compared with PART. This is in line with the progression of tauopathy to neocortical regions and likely with detrimental effects of tau and Aβ in the shared territorial brain areas of ADNC and PART. Interestingly, the volume of the entorhinal cortex was not found to be different between ADNC w/o LB and PART groups, supporting the possibility that, in PART, an initial tau‐initiated neurodegeneration might be in place.

Importantly, the distribution pattern of NFTs and LBs in PART+LB and ADNC+LB was different. With the increase in NP load, LB pathology progressed from the brainstem to neocortical regions, accompanying the evolution of NFT pathology across its Braak stages. This pattern has also been hinted at previously in a larger sample‐size study.^39^ This raises the hypothesis that the presence of NPs might be associated not only with the progression of tauopathy but also with the progression of LBs into neocortical stages. Interestingly, injection of alpha‐synuclein‐preformed fibrils in an amyloidogenesis mouse model exacerbates alpha‐synuclein inclusion pathology spread compared with a model without Aβ.^74^ Further mechanistic research is needed to assess this relationship.

As limitations of this study, the cohort is a convenience sample, since it was not created specifically to address the current questions. We were not able to add a “pure” LBD group as a control to better assess the potential additive effects of NFTs and LB pathology. LATE‐NC and CAA showed preferential accumulation among ADNC+LB groups, suggesting that higher co‐pathology tends to co‐occur together. Future studies aimed at disentangling the effects of these co‐pathologies among these groups will be pursued. Additionally, a higher sample size per group in this study could improve the generalizability of the MRI results.

In conclusion, this study shows differential patterns of dementia severity and cortical and subcortical atrophy in PART and ADNC with and without LB pathology. We provide evidence supporting PART+LB as a distinct group presenting with dementia severity progression intermediate between PART w/o LB and AD with and without LB pathology across multiple cognitive domains, patterns of brain atrophy, and with a topographical pattern of tau and alpha‐synuclein pathology that precedes that of ADNC. These results emphasize the growing importance of LB co‐pathology in AD‐related diseases with implications for clinical trial design and pathophysiological research.

## CONFLICT OF INTEREST STATEMENT

T.G.O. is a scientific advisor and shareholder of Ceracuity Inc., has been a consultant for Sonae and Guidepoint, and has received fees as a speaker from Eisai and conference fees covered from Roche. The remaining authors have no disclosures to report. Author disclosures are available in the Supporting Information.

## CONSENT STATEMENT

All participants provided written informed consent at each local ADC, and the study protocols were approved by the respective institutional review boards. The participants who contributed to the Neuropathology Data Set gave consent for autopsy. All participants provided written informed consent at each ADC.

## Supporting information

Supporting Information

Supporting Information

## Data Availability

The datasets used for the current study are available upon request at the NACC.
