# Supplementary material for: Lewy body co‐pathology in Alzheimer's disease and primary age‐related tauopathy contributes to differential neuropathological, cognitive, and brain atrophy patterns
Source: Alzheimers Dement. 2024 Dec 22;21(1):e14191. doi: 10.1002/alz.14191 (PMC11772724; doi:10.1002/alz.14191)
Supplement: Supplementary file 1 — Supporting Information [file ALZ-21-e14191-s001.docx]

**Supplementary Figure 1 – Participant selection flowchart**

**
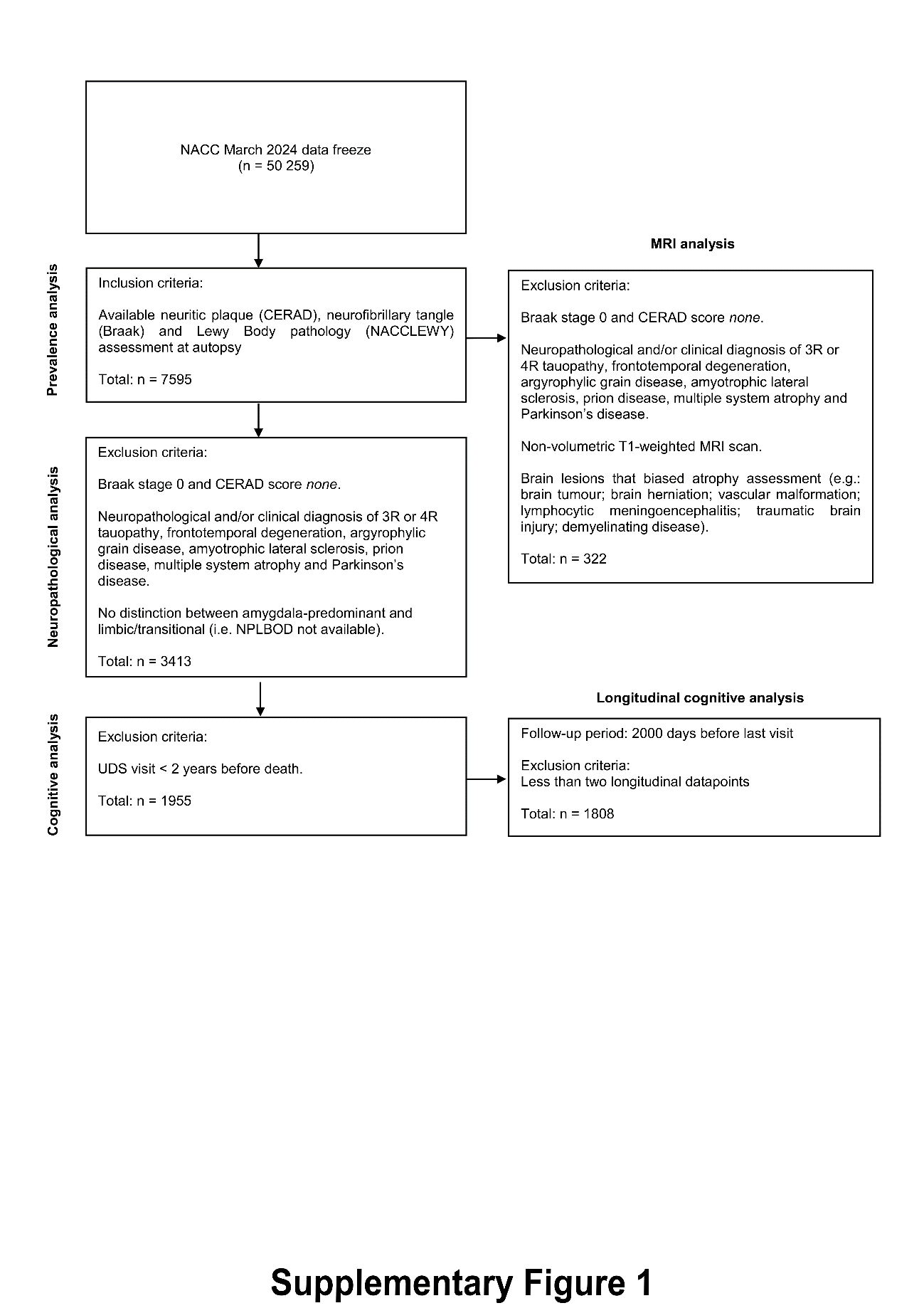
**

Supplementary Figure 1 shows the participant selection flowchart from the NACC cohort. Out of 50259 participants in the NACC March 2024 data freeze, 7595 with available neuritic plaque (CERAD), neurofibrillary tangle (Braak) and Lewy Body (LB) pathology (“NACCLEWY” variable) assessment at autopsy were included. For MRI analysis, participants with other neuropathologies, no ADNC neuropathology, without a volumetric T1-weighted acquisition or with brain lesions capable of biasing volumetric calculations were excluded, with a final sample of 322 participants. For detailed neuropathological analysis, after exclusion of participants with other neuropathologies, no ADNC neuropathology and no distinction of “amygdala-predominant” and limbic LB stages (without “NPLBOD” variable available), 3413 remained. For cross-sectional cognitive analysis, participants with a UDS visit more than 2 years before death were excluded, with 1955 remaining. For longitudinal cognitive analysis, a follow-up period of 2000 days before last visit was used and participants with less than two longitudinal data points were excluded. 1808 participants were included in this analysis.

**Supplementary Figure 2 – Prevalence of each group in the neuropathological cohort**

**
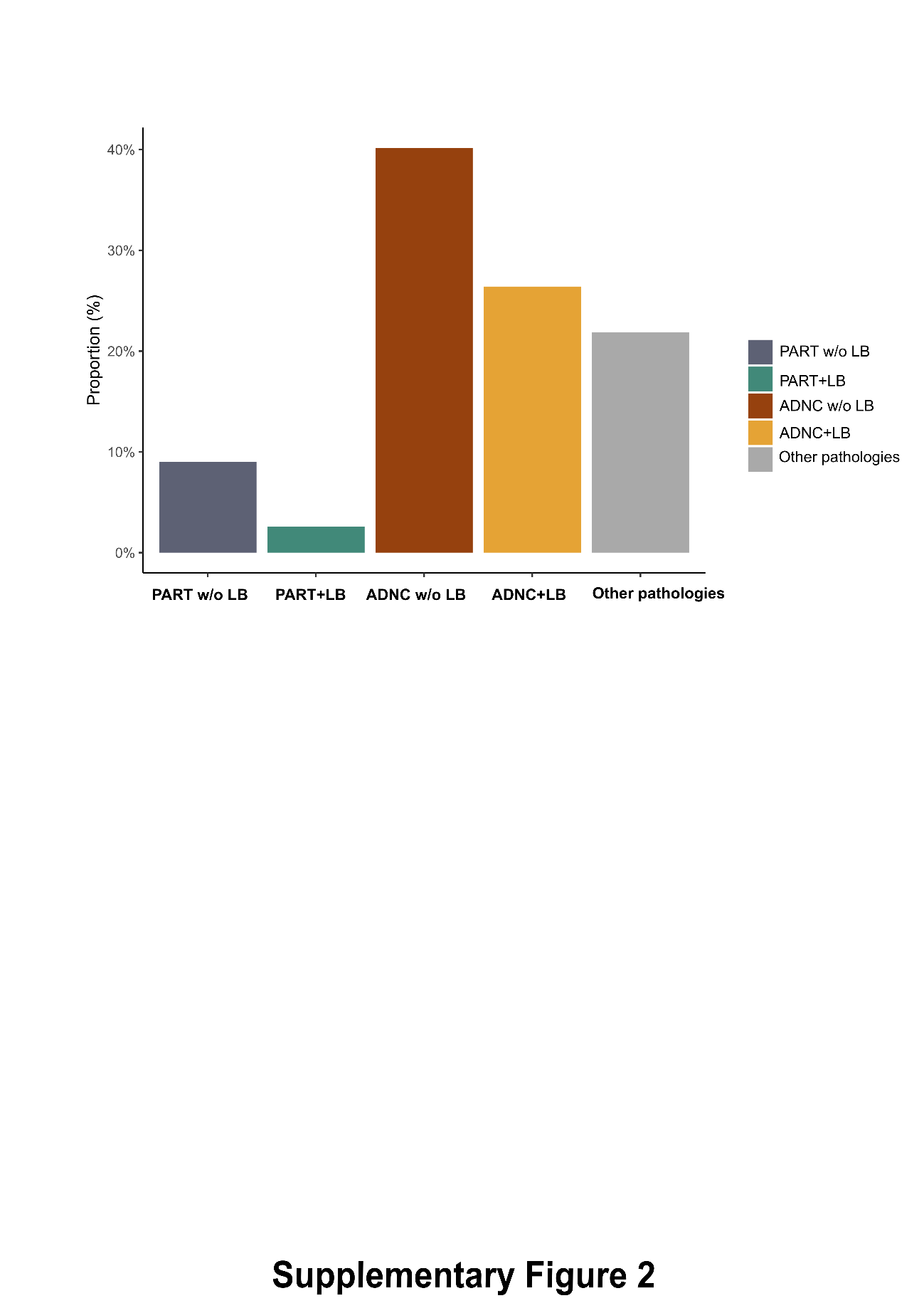
**

Supplementary Figure 2 shows the proportion of each neuropathological group in the prevalence cohort. ADNC w/o LB was the most frequent group (40.2%), followed by ADNC+LB (26.4%), other pathologies (21.8%), PART w/o LB (9.0%) and PART+LB (2.6%). Other pathologies corresponds to the presence of neuropathological or clinical evidence of 3R or 4R tauopathy, frontotemporal degeneration, argyrophylic grain disease, amyotrophic lateral sclerosis, prion disease, multiple system atrophy and Parkinson’s disease. Sample size, n = 7595 (PART w/o LB, n = 684; PART+LB, n = 195; ADNC w/o LB, n = 3051; ADNC+LB, n = 2006; Other pathologies, n = 1659).

**Supplementary Figure 3 – Lewy body co-pathology contributes to cognitive impairment independently of Braak stage**

**
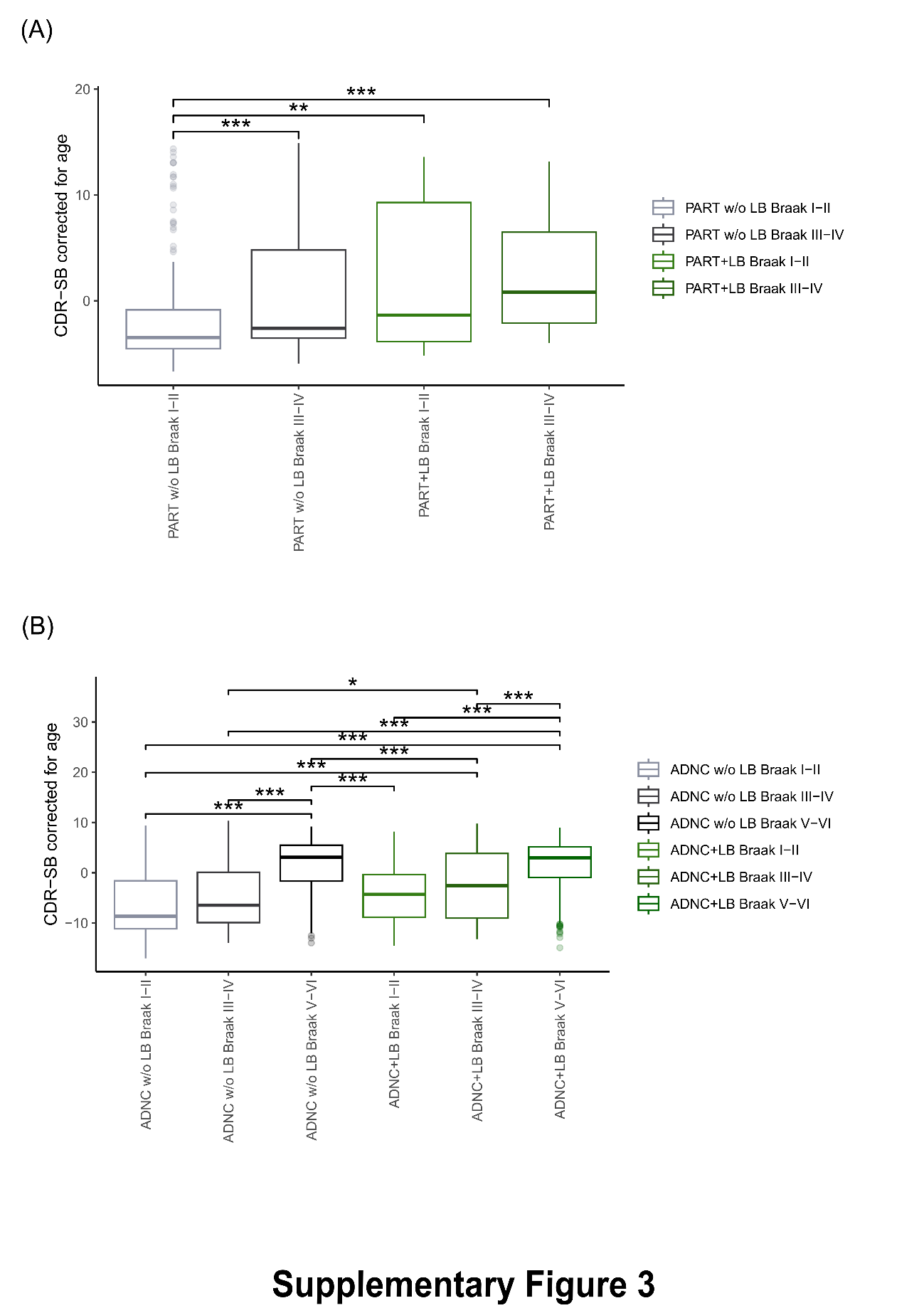
**

Supplementary Figure 3 shows (A) gradient increase of CDR-SB residuals after age correction with Braak stage and LB stage. Corrected CDR-SB was higher in PART+LB with Braak I-II and III-IV stages compared to PART w/o LB Braak I-II (p < 0.05 after Dunn’s test with Holm correction). Braak stage V-VI cases in both groups were excluded from the analysis due to low sample size. Sample size, n = 331 (PART w/o LB, Braak I-II, n = 173; PART w/o LB, Braak III-IV, n = 87; PART+LB, Braak I-II, n = 37; PART+LB, Braak III-IV, n = 34). (B) shows a similar analysis in ADNC groups, showing a gradient effect according to both Braak stage and LB stage. Note that ADNC+LB with Braak stage III-IV was significantly different from ADNC w/o LB with Braak stage III-IV. Sample size, n = 1612 (ADNC w/o LB, Braak I-II, n = 80; ADNC w/o LB, Braak III-IV, n = 224; ADNC w/o LB, Braak V-VI, n = 646; ADNC+LB, Braak I-II, n = 31; ADNC+LB, Braak III-IV, n = 93; ADNC+LB, Braak V-VI, n = 538). **p* < .05; ***p* < .01; ****p* < .001.

**Supplementary Figure 4 – Longitudinal progression of cognitive testing across groups**


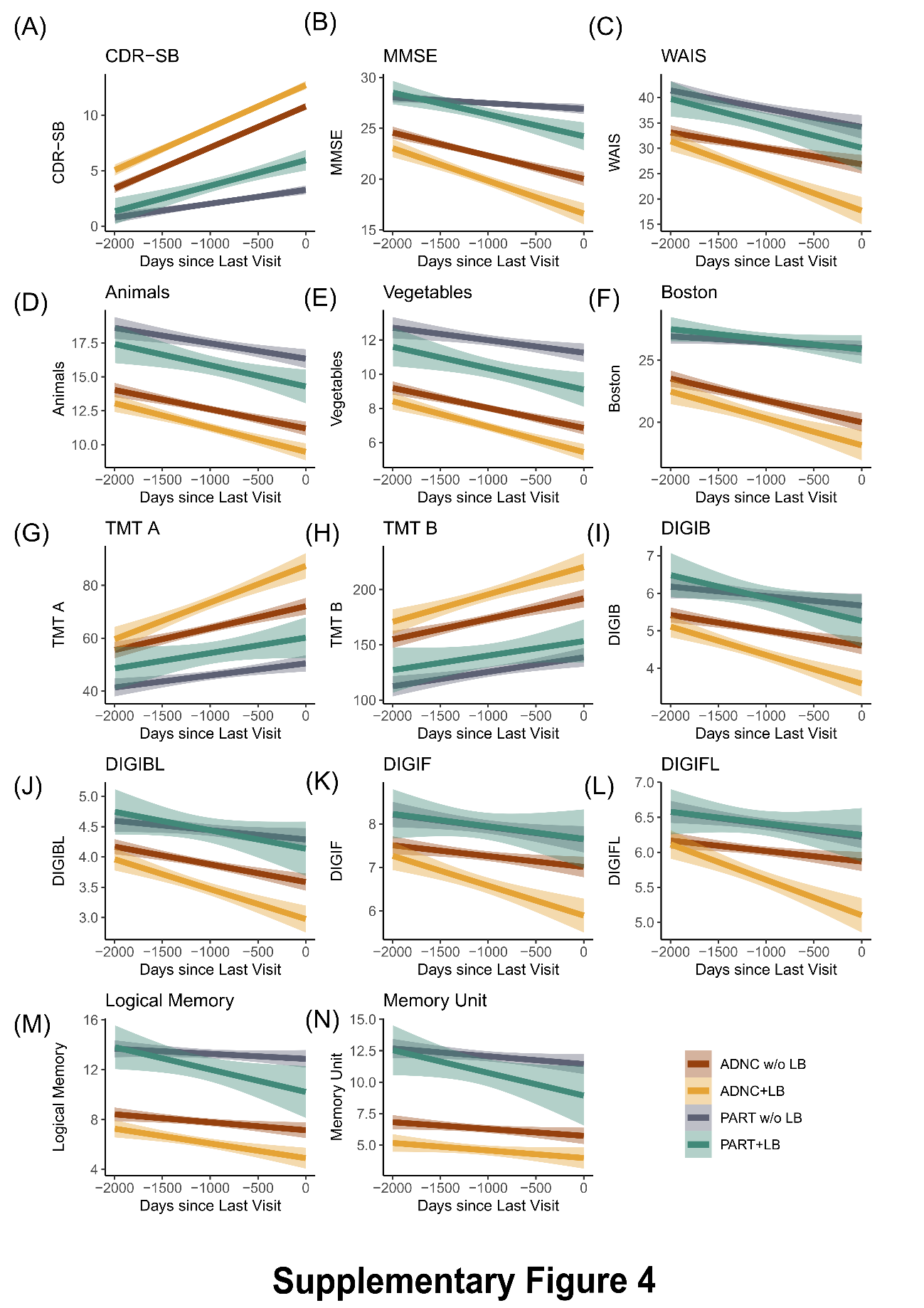


Supplementary Figure 4 shows the progression of cognitive impairment of each group across a range of neuropsychological tests (A-N). WAIS - Wechsler Adult Intelligence Scale; TMT – Trail Making Test; DIGIB – Digit Span Backwards; DIGIBL - Digit Span Backwards Length; DIGIF – Digit Span Forwards; DIGIFL – Digit Span Forwards Length. See Supplementary Table 3 for sample size.

**Supplementary Figure 5 – Correction for Braak stage shows regional associations of LB with regional atrophy in PART and ADNC**


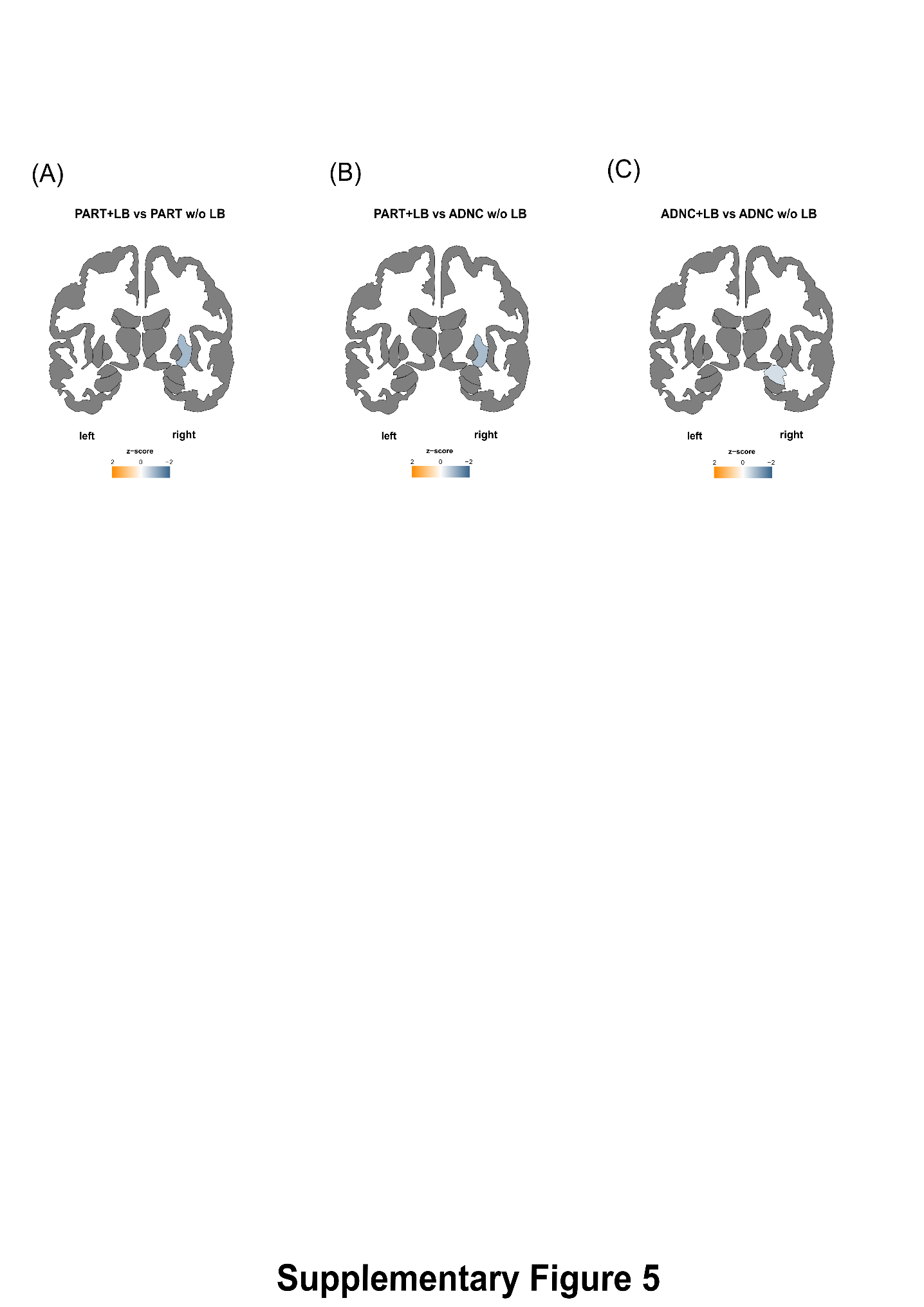


Supplementary Figure 5 shows the comparison of cortical and subcortical volumes for ADNC and PART with and without LBs. Z-scores based on mean difference in brain volume residuals after linear regression with age at MRI and Braak stage are shown in the brain graph in (A) – PART+LB versus PART w/o LB; (B) – PART+LB versus ADNC+LB; (C) – ADNC+LB versus ADNC w/o LB. The presence of LB pathology associated with lower right putamen volume in PART+LB compared to PART w/o LB and ADNC w/o LB and right amygdala volume in ADNC+LB compared to ADNC w/o LB. Blue regions represent lower volumes for the first group in the comparison. Only significantly different comparisons are shown (p < 0.05 after FDR correction for Welch’s ANOVA or Kruskall-Wallis Test and p < 0.05 after TukeyHSD or Dunn’s test with Holm correction). When not significant, group comparisons are not shown. Sample size, n = 1955 (PART w/o LB, n = 263; PART+LB, n = 75; ADNC w/o LB, n = 953; ADNC+LB, n = 664).

**Supplementary Tables**

**Supplementary Table 1 – Pairwise comparison p-values between ADNC and PART patients with and without Lewy bodies for demographic comparisons**

|  | PART w/o LB vs PART+LB | PART w/o LB vs ADNC w/o LB | PART w/o LB vs ADNC+LB | ADNC w/o LB vs PART+LB | ADNC w/o LB vs ADNC+LB | PART+LB vs ADNC+LB |
| --- | --- | --- | --- | --- | --- | --- |
| Sex proportions | **0.02** | 0.21 | 0.50 | **<0.01** | **<0.01** | **0.02** |
| Age at death | **0.05** | **<0.001** | **<0.01** | 0.31 | **<0.01** | **<0.01** |

Supplementary Table 1 – Pairwise comparisons between ADNC and PART patients with and without Lewy bodies for demographic characteristics. P-values are shown for each comparison using pairwise chi-square and Dunn’s test with Holm correction, respectively, for sex proportions and age at death.

**Supplementary Table 2 – Distribution of LATE-NC and CAA across groups**

| Cohort | PART w/o LB | PART+LB | ADNC w/o LB | ADNC+LB |  | p-value |
| --- | --- | --- | --- | --- | --- | --- |
| Sample | 264 | 86 | 1064 | 916 |  |  |
| LATE-NC (%) | 56 (21.2%) | 24 (27.9%) | 355 (33.4%) | 426 (46.5%) |  | ***X*(3) = 72.86;**  **p < 0.001** |
| Sample | 394 | 122 | 1613 | 1275 |  |  |
| CAA (%)  None  Mild  Moderate  Severe | 301 (76.4%)  61 (15.5%)  24 (6.1%)  8 (2.0%) | 87 (71.3%)  25 (20.5%)  7 (5.7%)  3 (2.5%) | 403 (25.0%)  561 (34.8%)  426 (26.4%)  223 (13.8%) | 223 (17.5%)  450 (35.3%)  381 (29.9%)  221 (17.3%) |  | ***X*(9) = 634.7;**  **p < 0.001** |
| Sample | 101 | 32 | 318 | 271 |  |  |
| ARTAG (%) | 45 (44.6%) | 17 (53.1%) | 125 (39.3%) | 116 (42.8%) |  | *X(3) = 2.91;* p = 0.40 |

Supplementary Table 2 – Distribution of LATE-NC, CAA and ARTAG proportions in ADNC and PART patients with and without Lewy bodies. Groups differed in frequency of cases with LATE-NC and CAA. Both CAA and Late-NC were more frequent in ADNC+LB. P-values are shown for each comparison using chi-square tests. Pairwise comparisons showed higher proportion of LATE-NC in ADNC+LB compared to PART-LB and PART+LB, higher proportion of CAA in ADNC+LB and ADNC w/o LB compared to both PART w/o LB and PART+LB (p-value < 0.05 with chi-square tests after Holm correction).

**Supplementary Table 3 – Median visits, interquartile range and sample size per group according to neuropsychological test availability across the longitudinal follow-up period.**

|  | PART w/o LB | PART+LB | ADNC w/o LB | ADNC+LB |
| --- | --- | --- | --- | --- |
| CDR-SB [IQR] | 5 [2]; n = 245 | 5 [3]; n = 71 | 5 [2]; n = 893 | 5 [3]; n = 599 |
| MMSE  [IQR] | 4 [3]; n = 191 | 3 [3]; n = 52 | 3 [2]; n = 596 | 2 [3]; n = 363 |
| WAIS  [IQR] | 3 [2]; n = 178 | 2 [3]; n = 49 | 2 [3]; n = 516 | 2 [2]; n = 302 |
| Animals  [IQR] | 5 [2]; n = 233 | 4 [3]; n = 65 | 3 [3]; n = 737 | 3 [2]; n = 469 |
| Vegetables  [IQR] | 5 [2]; n = 233 | 4 [3]; n = 65 | 3 [3]; n = 737 | 3 [2]; n = 469 |
| Boston  [IQR] | 4 [3]; n = 188 | 3 [3]; n = 52 | 3 [2]; n = 577 | 2 [3]; n = 341 |
| TMT A  [IQR] | 5 [3]; n = 229 | 4 [3]; n = 65 | 3 [3]; n = 681 | 2 [3]; n = 430 |
| TMT B  [IQR] | 4 [3]; n = 222 | 4 [3]; n = 61 | 3 [2]; n = 591 | 2 [3]; n = 348 |
| DIGIB  [IQR] | 4 [3]; n = 188 | 3 [3]; n = 52 | 3 [2]; n = 580 | 2 [3]; n = 343 |
| DIGIBL  [IQR] | 4 [3]; n = 188 | 3 [3]; n = 52 | 3 [2]; n = 580 | 2 [3]; n = 343 |
| DIGIF  [IQR] | 4 [3]; n = 188 | 3 [3]; n = 52 | 3 [2]; n = 580 | 2 [3]; n = 343 |
| DIGIFL  [IQR] | 4 [3]; n = 188 | 3 [3]; n = 52 | 3 [2]; n = 580 | 2 [3]; n = 343 |
| Logical Memory  [IQR] | 4 [3]; n = 188 | 3 [3]; n = 51 | 3 [2]; n = 578 | 2 [3]; n = 338 |
| Memory Unit  [IQR] | 4 [3]; n = 188 | 3 [3]; n = 51 | 3 [2]; n = 573 | 2 [3]; n = 337 |

Supplementary Table 2 – Median visits, interquartile range and sample size per group according to neuropsychological test availability across the longitudinal follow-up period. MMSE – mini-mental state examination; WAIS - Wechsler Adult Intelligence Scale; TMT – Trail Making Test; DIGIB – Digit Span Backwards; DIGIBL - Digit Span Backwards Length; DIGIF – Digit Span Forwards; DIGIFL – Digit Span Forwards Length; IQR – interquartile range.

**Supplementary Table 4 – Beta-coefficients, standard error and p-values for multiple linear regression mixed-effects models with cognitive testing**

|  | Intercept | PART+LB | ADNC w/o LB | ADNC+LB | Time | PART+LB *Time | ADNC w/o LB * Time | ADNC+LB * Time |
| --- | --- | --- | --- | --- | --- | --- | --- | --- |
| CDR-SB [SE] | 3.29***  [0.41] | **2.76****  **[0.86]** | **7.54*** [0.46]** | **9.68*****  **[0.48]** | **2.41*** [0.41]** | **2.54** [0.88]** | **5.60*** [1.56]** | **6.61*****  **[0.49]** |
| MMSE  [SE] | 26.17*** [0.81] | -2.57* [1.78] | **-10.76*** [0.94]** | **-14.56*** [1.03]** | **-1.64* [0.72]** | -2.53 [1.61] | **-8.45*** [0.84]** | **-10.86*** [0.94]** |
| WAIS  [SE] | 34.01*** [1.49] | **-7.48* [3.31]** | **-14.64*** [1.76]** | **-23.83*** [1.98]** | **-6.13*****  **[1.40]** | -4.96 [3.20] | **-8.22*** [1.68]** | **-15.38*** [1.97]** |
| Animals  [SE] | 15.56*** [0.48] | **-2.37* [1.04]** | **-7.40*** [0.56]** | **-9.40*** [0.61]** | **-2.85*** [0.44]** | -1.09 [0.98] | **-3.41*** [0.53]** | **-4.90*** [0.61]** |
| Vegetables  [SE] | 10.62*** [0.35] | **-2.38** [0.76]** | **-5.83*****  **[0.41]** | **-7.33*** [0.45]** | **-2.07*** [0.33]** | -1.08 [0.76] | **-2.67*** [0.41]** | **-3.40*** [0.47]** |
| Boston  [SE] | 25.99*** [0.89] | -0.32 [1.93] | **-10.27*** [1.03]** | **-13.36*****  **[1.14]** | -0.64 [0.75] | -0.75 [1.69] | **-7.94*** [0.89]** | **-10.11*****  **[1.01]** |
| TMT A  [SE] | 55.66*** [3.70] | **23.22*** [7.96]** | **40.69*** [4.36]** | **57.33*****  **[4.78]** | **13.73*** [3.42]** | **22.04** [7.62]** | **31.43*** [4.15]** | **45.71*** [4.70]** |
| TMT B  [SE] | 156.75*** [7.13] | **37.10* [15.84]** | **89.67*****  **[8.66]** | **122.39*****  **[9.84]** | **43.99*** [7.14]** | 24.83  [16.46] | **53.10*****  **[8.98]** | **75.64*****  **[10.70]** |
| DIGIB  [SE] | 5.55*** [0.21] | -0.66 [0.47] | **-1.77*** [0.24]** | **-2.80*****  **[0.28]** | **-0.51* [0.22]** | -0.90 [0.53] | **-1.22*** [0.27]** | **-1.87*****  **[0.32]** |
| DIGIBL  [SE] | 4.21*** [0.13] | -0.26 [0.31] | **-1.14*** [0.16]** | **-1.80*****  **[0.18]** | -0.29  [0.15] | -0.40 [0.35] | **-0.87*** [0.18]** | **-1.26*****  **[0.21]** |
| DIGIF  [SE] | 7.68*** [0.23] | -0.63  [0.52] | **-1.59*** [0.27]** | **-2.79*****  **[0.30]** | **-0.51***  **[0.23]** | -0.71  [0.54] | **-1.11*****  **[0.28]** | **-1.91*****  **[0.32]** |
| DIGIFL  [SE] | 6.21*** [0.15] | -0.24  [0.34] | **-0.85*****  **[0.17]** | **-1.67*****  **[0.20]** | **-0.35***  **[0.15]** | -0.26  [0.36] | **-0.54****  **[0.18]** | -1.20***  [0.21] |
| Logical Memory  [SE] | 12.46*** [0.50] | -1.86 [1.13] | **-7.57*** [0.59]** | **-9.77*****  **[0.66]** | -0.47 [0.45] | -1.18  [1.06] | **-2.99*****  **[0.55]** | **-4.18*** [0.63]** |
| Memory Unit  [SE] | 11.05*** [0.49] | -1.75  [1.10] | **-7.01*** [0.57]** | **-8.91*****  **[0.64]** | -0.73 [0.44] | -0.77 [1.05] | **-1.85*****  **[0.54]** | **-2.40*****  **[0.62]** |

Supplementary Table 3 – Beta-coefficients, standard error and p-values for multiple linear regression mixed-effects models with cognitive testing. MMSE – mini-mental state examination; WAIS - Wechsler Adult Intelligence Scale; TMT – Trail Making Test; DIGIB – Digit Span Backwards; DIGIBL - Digit Span Backwards Length; DIGIF – Digit Span Forwards; DIGIFL – Digit Span Forwards Length; SE - Standard error; *p < .05; **p < .01; ***p < .001.

**Supplementary Table 5 – Estimates, standard errors and p-values for marginal slope pairwise comparisons.**

|  | PART w/o LB vs PART+LB | PART w/o LB vs ADNC w/o LB | PART w/o LB vs ADNC+LB | PART+LB vs ADNC w/o LB | PART+LB vs ADNC+LB | ADNC w/o LB vs ADNC+LB |
| --- | --- | --- | --- | --- | --- | --- |
| CDR-SB [SE] | **-2.55* [0.88]** | **-5.60*** [0.47]** | **-6.62*** [0.49]** | **-3.06** [0.81]** | **-4.07*** [0.83]** | **-1.01* [0.35]** |
| MMSE  [SE] | 2.53 [1.61] | **8.45*** [0.84]** | **10.86*** [0.94]** | **5.92*** [1.50]** | **8.33*** [1.56]** | **2.41*** [0.75]** |
| WAIS  [SE] | 4.96 [3.21] | **8.22*** [1.68]** | **15.38*** [1.97]** | 3.26 [3.03] | **10.42** [3.20]** | **7.16*** [1.67]** |
| Animals  [SE] | 1.09 [0.98] | **3.41*** [0.53]** | **4.90*** [0.61]** | 2.32 [0.93] | **3.81*** [0.98]** | **1.49* [0.52]** |
| Vegetables  [SE] | 1.08 [0.75] | **2.66*** [0.40]** | **3.39*** [0.46]** | 1.58 [0.71] | **2.31* [0.75]** | 0.73 [0.39] |
| Boston  [SE] | 0.76 [1.69] | **7.93*** [0.89]** | **10.10*** [1.01]** | **7.16*** [1.59]** | **9.33*** [1.66]** | **2.17* [0.82]** |
| TMT A  [SE] | **-22.04* [7.62]** | **-31.43*** [4.15]** | **-45.71*****  **[4.70]** | -9.39 [7.21] | **-23.67****  **[7.54]** | **-14.27** [4.00]** |
| TMT B  [SE] | -24.80 [16.47] | **-53.10***[8.98]** | **-75.60*****  **[10.71]** | -28.30 [15.81] | **-50.80***  **[16.85]** | -22.50 [9.67] |
| DIGIB  [SE] | 0.90 [0.53] | **1.22*** [0.27]** | **1.87*** [0.32]** | 0.32 [0.51] | 0.96 [0.53] | 0.64 [0.27] |
| DIGIBL  [SE] | 0.40 [0.35] | **0.87*** [0.18]** | **1.26*** [0.21]** | 0.46 [0.33] | 0.86 [0.35] | 0.39 [0.17] |
| DIGIF  [SE] | 0.71 [0.54] | **1.11*** [0.28]** | **1.91*** [0.32]** | 0.40 [0.51] | 1.20 [0.54] | **0.79* [0.27]** |
| DIGIFL  [SE] | 0.26 [0.36] | **0.54* [0.18]** | **1.20*** [0.21]** | 0.28 [0.34] | **0.94* [0.36]** | **0.65** [0.18]** |
| Logical Memory  [SE] | 1.19 [1.06] | **2.99*** [0.55]** | **4.18*** [0.63]** | 1.81 [1.01] | **2.99* [1.06]** | 1.19 [0.54] |
| Memory Unit  [SE] | 0.77 [1.05] | **1.85** [0.54]** | **2.40*** [0.62]** | 1.08 [0.99] | 1.63 [1.04] | 0.55 [0.53] |

Supplementary Table 5 – Estimates, standard errors and p-values for marginal slope pairwise comparisons. MMSE – mini-mental state examination; WAIS - Wechsler Adult Intelligence Scale; TMT – Trail Making Test; DIGIB – Digit Span Backwards; DIGIBL - Digit Span Backwards Length; DIGIF – Digit Span Forwards; DIGIFL – Digit Span Forwards Length; SE – Standard error; *p < .05; **p < .01; ***p < .001.
